# Kinetics and product identification of water-dissolved nitroguaiacol photolysis under artificial sunlight

**DOI:** 10.3389/fchem.2023.1211061

**Published:** 2023-07-14

**Authors:** Ajda Delić, Urša Skube, Martin Šala, Ana Kroflič

**Affiliations:** ^1^ Department of Catalysis and Chemical Reaction Engineering, National Institute of Chemistry, Ljubljana, Slovenia; ^2^ Department of Analytical Chemistry, National Institute of Chemistry, Ljubljana, Slovenia

**Keywords:** 4-nitroguaiacol, 5-nitroguaiacol, 2-methoxyphenol, secondary organic aerosol, photodegradation, atmospheric aqueous phase, atmospheric lifetime, brown carbon

## Abstract

Nitroguaiacols are typical constituents of biomass-burning emissions, including absorbing aerosols which contribute to climate change. Although they are also harmful to humans and plants, their atmospheric fate and lifetimes are still very speculative. Therefore, in this work, the photolysis kinetics of aqueous-phase 4-nitroguaiacol (4NG) and 5-nitroguaiacol (5NG), and the resulting photo-formed products were investigated under artificial sunlight, observing also the effect of sunlight on the absorption properties of the solutions. We found the photolysis of 5NG slower than that of 4NG, whereas the absorbance in the visible range prevailed in the 5NG solutions at the end of experiments. Although we identified dinitroguaiacol as one of the 4NG photolysis products, which increased light absorption of 4NG-containing solutions, considerably more chromophores formed in the 5NG photolyzed solutions, implying its stronger potential for secondary BrC formation in the atmosphere. In general, denitration, carbon loss, hydroxylation, nitration, and carbon gain were characteristic of 4NG phototransformation, while carbon loss, hydroxylation, and carbon gain were observed in the case of 5NG. The photolysis kinetics was found of the first order at low precursor concentrations (<0.45 mM), resulting in their lifetimes in the order of days (125 and 167 h illumination for 4NG and 5NG, respectively), which suggests long-range transport of the investigated compounds in the atmosphere and proposes their use as biomass-burning aerosol tracer compounds.

## 1 Introduction

Nitrophenols (NPs, Ph-NO_2_), i.e., nitrated compounds with the phenolic moiety in their structure, are common constituents of ambient air with known phytotoxic activity. In particular, NPs attract our attention because in the past, they have been linked with remote forest decline (Rippen et al., 1987; Natangelo et al., 1999), while eco- and cytotoxicity of NPs have also been confirmed in more recent studies (Subashchandrabose et al., 2012; Pflieger and Kroflič, 2017; Majewska et al., 2021). Moreover, particulate NPs, such as 4-nitrophenol, 4-nitrocatechol, methylnitrophenols, and nitroguaiacols (NGs) that are closely connected with lignin biomass burning have been shown predominant constituents of atmospheric brown carbon (BrC, near-UV and visible light-absorbing component of atmospheric aerosols), giving atmospheric aerosols typical yellow color and contributing to climate change by light absorption and subsequent warming of the atmosphere (Li et al., 2021).

Airborne NPs can be directly emitted into the atmosphere during various types of fuel combustion or form in secondary atmospheric processes from diverse aromatic precursor gases (Roger et al., 1992; Harrison et al., 2005; Kroflič et al., 2021). In general, NPs are semi-volatile in their nature, therefore they tend to partition between different atmospheric compartments, *i.e.*, gaseous, liquid and particulate phases (Sakakibara et al., 2022), each of them acting as a specific chemical reactor in which NPs interact with different reactive species and sunlight. Due to the chemical stability of NPs compared with their non-nitrated analogues (Grosjean, 1991), their residence time in the atmosphere is generally considered long, which intuitively implies long traveling distances from their emission sources (Kroflič et al., 2015a). However, especially photostability still needs to be evaluated for specific groups of NP compounds as photolysis kinetics and atmospheric lifetimes of many NPs are missing.

To date, environmentally relevant studies of nitrophenol photolysis have mostly focused on the formation of gaseous nitrous acid (HONO), which is the major source of hydroxyl radicals (^•^OH) in the troposphere during early morning. Already 2 decades ago, direct photolysis of gas-phase *ortho*-nitrophenol (*o*-NP) was identified as a potential important source of ambient HONO, with possible further implications in the oxidative capacity of the atmosphere (Bejan et al., 2006). Later on, this photolysis reaction has been investigated in many laboratory (Bejan et al., 2007; Wei et al., 2008; Sangwan and Zhu, 2018), theoretical (Vereecken et al., 2016; Guo and Li, 2022), and combined experimental-theoretical studies (Cheng et al., 2009; Nitta et al., 2021), which further suggested that besides nitrogen release, ^•^OH can also form directly during the photolytic degradation of gaseous *o*-NP, resulting in aromatic nitroso products. In a recent field study conducted in an urban environment, however, a minor role has been attributed to this second pathway (Cheng et al., 2021). Moreover, very recently, photolysis frequency of selected gas-phase NPs has been estimated from ambient measurements, resulting in their atmospheric lifetimes in the order of minutes (Peng et al., 2023), which contradicts the general belief that they are long-lived in the atmosphere. Finally, it has been shown that direct photolysis is the dominant degradation pathway of gas-phase *o*-NP in the atmosphere, corresponding to the photolysis lifetimes in the order of minutes compared with the estimated atmospheric ^•^OH radical reaction lifetime of 193 h (Sangwan and Zhu, 2016). Although studies on gaseous NPs other than unsubstituted phenols are scarce, knowledge on the photolysis kinetics and mechanisms of environmentally relevant semi-volatile NPs in the atmospheric condensed phase is even poorer.

In contrast to the gas-phase photolysis pathways just described, it has been recently shown that *para*-nitrophenol (*p*-NP) photolyses more rapidly than the other nitrophenol isomers in aqueous solutions and viscous aqueous films, resulting in HONO and/or nitrite build-up similarly to the gas-phase *o*-NP (Barsotti et al., 2017). Chen and co-workers (2005) studied direct photolysis of all three nitrophenol isomers in aqueous solutions. Among the most important pathways leading to the observed photoproducts, excited triplet state formation followed by nitro-nitrite rearrangement was proposed, yielding the corresponding phenoxy radical and NO after the O–NO bond cleavage (Chen et al., 2005). In addition, the second pathway of photoinduced aqueous-phase nitrophenol denitration accompanied by the HONO release has been studied by several authors (Chen et al., 2005; Barsotti et al., 2017; Guo and Li, 2022). Different mechanisms have been proposed for this step, yielding whole range of speculative reaction products. Alternatively, NO and NO_2_ cannot be excluded as the ring-leaving groups as well (Barsotti et al., 2017; Guo and Li, 2022). Despite a few mechanistic studies conducted, to the best of our knowledge photolysis rates and/or atmospheric lifetimes of aqueous-phase NPs when exposed to sunlight are completely unknown.

Although guaiacol (2-methoxyphenol, GUA) is a common model compound for biomass-burning emissions, which is known to produce substantial amounts of toxic NG chromophores under specific conditions in the atmosphere (Kroflič et al., 2021), the photodegradation kinetics of its secondary nitrated products is still speculative in the literature (Iinuma et al., 2007; Lauraguais et al., 2014). Different aqueous-phase transformations of GUA under various atmospherically-relevant conditions have already been studied (Kroflič et al., 2015b; Slikboer et al., 2015; Kroflič et al., 2018; Yang et al., 2021), whereas atmospheric lifetimes of the formed NGs in a sense of their resistance to direct photolysis have never been determined to date. To elucidate the fate of biomass-burning NGs in the atmospheric waters, aqueous solutions containing 4- and 5-nitroguaiacol (4NG and 5NG, respectively) as atmospherically abundant NGs were investigated upon illumination by a solar simulator. The aim of the study was to evaluate the kinetics of their direct photolysis and identify the main products that retain the aromatic moiety at the end of experiment. This further allowed us to determine daytime atmospheric lifetimes due to aqueous-phase photolysis of the investigated compounds and identify characteristic pathways of direct NG phototransformations, which are summarized in schematic representations underlying primary and secondary reaction products formation.

## 2 Materials and methods

### 2.1 Materials

For the preparation of standard solutions and reaction mixtures, GUA (2-methoxyphenol, CAS: 90-05-1, Sigma), 4NG (2-methoxy-4-nitrophenol, CAS: 3251-56-7, Sigma Aldrich), 5NG (2-methoxy-5-nitrophenol, CAS: 636-93-1, Fluka), 4,6-dinitroguaiacol (46DNG; 2-methoxy-4,6-dinitrophenol, CAS: 4097-63-6, AKos GmbH) and 4-nitrocathecol (4NC; 4-nitrobenzene-1,2-diol, CAS: 3316-09-4, Fluka) were used. Their formulas and the corresponding deprotonated molecular ions are collected in Supplementary Table S1. The purity of all standard compounds was ≥95%. Standard solutions and reaction mixtures were prepared with high-purity water (18.2 MΩ·cm) supplied by a Milli-Q water purification system.

Standard buffer solutions (pH 10, pH 7, pH 4, and pH 2) were used for two-point pH meter calibration and to preliminary define the measurement range for the spectrophotometric determination of pKa values. Sodium hydroxide (NaOH, 0.01 M and 0.1 M) and/or sulfuric acid (95%, H_2_SO_4_, 0.09 M) were used for the pH adjustment of standard solutions.

For HPLC analyses, methanol (≥99.9%, Chromasolv for HPLC) and formic acid (98%–100%) were used for mobile phase preparation, while methanol (≥99.9%, Chromasolv for LC/MS) and formic acid (98%–100%, LiChropur) were used for the unknown products identification with HPLC-MS/MS.

### 2.2 Methods

#### 2.2.1 Photolysis experiments

Photolysis of diluted solutions containing 4NG and 5NG was carried out in a custom-made reactor using a LOT-Quantum Design Europe solar simulator equipped with an ozone-free Xenon short arc lamp operated at 250 W. Reaction mixtures at different initial concentrations were held at 19 cm from the light source in a round bottom flask made from the borosilicate DURAN^®^ glass, partially submerged in a thermostated water bath at 25°C and thoroughly mixed by rotation. According to the instrument specifications, the produced irradiation was equivalent to approximately one sun (∼1000 W/m^2^). Note at this point that DURAN^®^ glass substantially absorbs light only below about 300 nm, which is comparable with stratospheric ozone, mimicking real atmospheric conditions. Emission spectrum of the Xenon lamp above and under the glass of the reactor flask together with the absorption spectra of 4NG and 5NG is shown in Supplementary Figure S1.

As atmospheric waters are mostly acidic due to the presence of dissolved CO_2_ and other water-soluble acids [pH values between 1.95 and 7.74 have been reported according to Vidović et al. (2018)], all starting reaction mixtures had their pH adjusted to around 5 with H_2_SO_4_. Each reaction mixture was kept under the light for at least 48 h, aliquots were taken at scheduled times and analyzed with a spectrophotometer and HPLC-UV/VIS-(MS/MS). Solution pH at the beginning and at the end of each experiment are given in Supplementary Table S2. Starting solutions of 4NG and 5NG were all stable when kept in dark (an example of blank experiments is shown in Supplementary Figure S2).

#### 2.2.2 Spectroscopic characterization of standard compounds and reaction mixtures

Spectroscopic measurements were performed with a Perkin Elmer Lambda 25 spectrophotometer with 1 nm resolution. Absorption spectra of standard solutions and reaction mixtures were recorded in the 200–700 nm range.

To assure that ionization of the studied phenolic compounds did not affect absorption spectra of the reaction mixtures, pKa values of 4NG, 5NG, 46DNG, and GUA were determined prior to the photolysis experiments. Conditions used for the determination of pKa values are collected in Supplementary Table S2. Small amounts of NaOH were consecutively added to the standard solutions in H_2_SO_4_ in the investigated pH range. At each step, pH of the solution was measured with a pH meter (Iskra pH meter MA 5736) while moderately stirring and a sample aliquot was taken for the absorbance measurement at the wavelength specified in Supplementary Table S3. The influence of sole NaOH and H_2_SO_4_ at the maximum added concentration was also tested and no absorbance of the investigated solvents at the characteristic wavelengths for model compounds was observed.

To obtain the pKa of each compound, the acquired absorbance data were plotted against the corresponding pH and fitted by a model function shown in Eq. 4. The model function was derived from the Henderson–Hasselbalch Eq. 1 and the Beer-Lamber law (Eq. 2) by considering additivity of absorbances and the mass balance equation (Eq. 3; *i* denotes protonated (HA) and deprotonated (A⁻) molecular forms).

$$pH={pK}_{a}+\log\frac{A^{-}}{HA}$$ (1)

$$A=\varepsilon \times l\times c=\sum A_{i}=\sum \varepsilon_{i}\times l\times c_{i}$$ (2)

$$c_{tot}=\sum c_{i}$$ (3)

$$A=A_{HA}-\frac{10^{pH-{pK}_{a}}}{1+10^{pH-{pK}_{a}}}\times \left(A_{HA}-A_{A^{-}}\right)$$ (4)

Three fitting parameters were always adjusted to give the lowest chi-square: absorbance of the completely protonated molecule (A_HA_), absorbance of the completely deprotonated molecule ( $A_{A^{-}}$ ), and pKa value of the compound.

#### 2.2.3 Product studies

Concentrations of target compounds in the reaction mixture aliquots were determined with a Thermo Scientific Dionex Ultimate 3000 RS UHPLC system equipped with a diode-array detector (DAD) with the adaptation of the previously developed method (Frka et al., 2016). Sample components were separated on an Atlantis T3 column (3.0 × 150 mm, 3 μm particle size, Waters) at 30°C by an isocratic elution with a mobile phase consisting of methanol/water (50/50, v/v) containing 0.1% (v/v) formic acid at a flow rate of 0.4 mL/min and detected with a DAD detector at 275 nm (GUA), 345 nm (4NG and 5NG), and 380 nm (46DNG). After the separation, unknown peaks were further investigated with a mass spectrometer (4000 QTRAP system Applied Biosystems/MDS Sciex) by using negative polarity electrospray ionization [(−)ESI]. For identification and quantification purposes, MS/MS and selected reaction monitoring (SRM) experiments were performed. The parameters used in SRM experiments are listed in Supplementary Table S4, whereas the declustering potential (DP) and the collision energy (CE) for the MS/MS experiments were selected in a similar range.

#### 2.2.4 Atmospheric lifetimes

To determine the atmospheric lifetime (τ) of a substance, changes in chemical composition with time are important. The collected kinetic data were thus studied and most suitable kinetic laws were determined for each photolysis experiment using the initial rate method.

The atmospheric lifetime is defined as the time in which investigated compound’s concentration drops to the 1/e of its original value, therefore it can only be determined for the first-order reactions (Eq. 5) as is shown in Eq. 6.

$$\ln\frac{\left[NG\right]}{{\left[NG\right]}_{0}}=-k_{1}t$$ (5)

$$\tau =\frac{1}{k_{1}}$$ (6)

For the zero-order reactions (Eq. 7), universal τ cannot be defined because it always depends on the initial pollutant concentration.

$$\left[NG\right]={\left[NG\right]}_{0}-k_{0}t$$ (7)

In the above equations, [NG]_0_ and [NG] stand for compound’s concentrations at the beginning and at a certain time *t* of the reaction, respectively, and *k*_0_ and *k*_1_ are the zero-order and first-order kinetic rate constants, respectively.

## 3 Results and discussion

### 3.1 Photo-spectroscopic characterization of standard solutions

**FIGURE 1:**
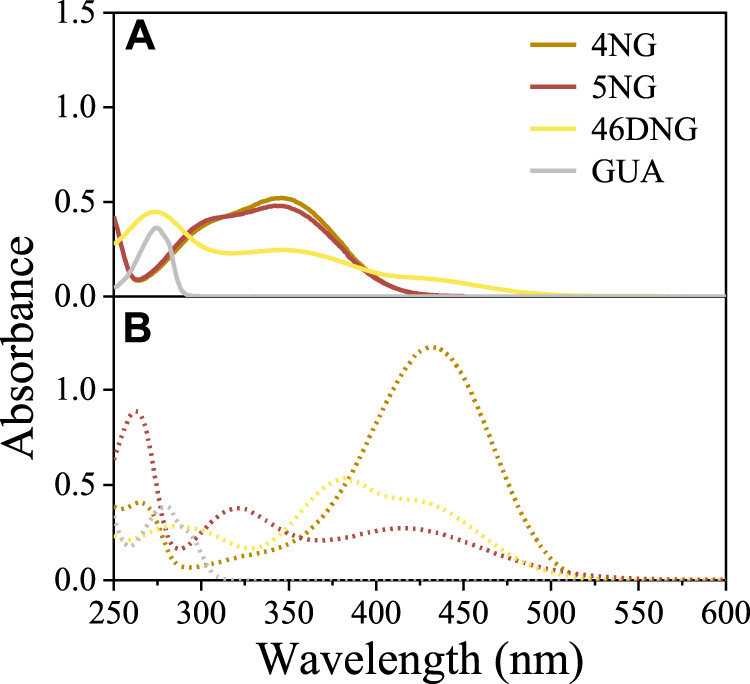
Light-absorption spectra of 4NG, 5NG, 46DNG, and GUA solutions at **(A)** pH 2 (solid lines) and **(B)** pH 10 (dotted lines).

Guaiacol only absorbs UV light (<310 nm) and the spectrum measured under acidic conditions (Figure 1A) compares very well with those found in the literature (Wang et al., 2019). On the other hand, NGs significantly absorb near UV and visible radiation (<450 nm and <500 nm for mono and dinitro compounds, respectively; Figure 1A), giving them yellow color characteristic of phenolic substances with nitro substituents. As the intensity of the color depends on the number and position of substituent nitro groups on the phenolic ring, especially 46DNG aqueous solutions are intensely yellow to brownish in color.

Nevertheless, besides the intrinsic chemical characteristics responsible for molecule’s visible light absorption (i.e., chromophores), pH of the solution can also influence the absorption spectrum of a compound with specific structural features (e.g., possessing acidic phenolic group; Figure 1). Exploring the effect of pH on the spectroscopic behavior of nitroguaiacols indeed gave us important results. At pH 2, typical absorbance bands in the near UV region (280–400 nm) were characteristic of methoxyphenols with one nitro group (i.e., 4NG and 5NG), similar to their analogous *m*-nitrophenol (*m*-NP) and *o*-NP (Bailey-Darland et al., 2023), while 46DNG significantly absorbed light also in the visible part of the spectrum (above 400 nm) (Figure 1A). However, the deprotonated forms of all investigated compounds at pH 10 exhibited significant absorption in the visible range of the spectrum (Figure 1B).

By increasing the solution pH, a shift of characteristic absorbance bands towards longer wavelengths was observed (Figure 1) and a more intense yellow color appeared. Similar has been observed for monosubstituted nitrophenol isomers (Bailey-Darland et al., 2023) and the bathochromic effect of pH on the spectroscopic properties has also been reported for real atmospheric samples with high content of aromatic components (Mo et al., 2017). This is typically attributed to the dissociation of a proton from the acidic phenolic hydroxy group.

From our pH-dependent spectroscopic data shown in Supplementary Figure S3 we were further able to determine pKa values of the investigated compounds, which are lacking in the current literature. The determined pKa values are increasing in the following order: 46DNG < 4NG < 5NG < GUA. To elucidate the influence of the position of the nitro group on the pKa of nitrated GUA, we compare those to the pKa values of a series of nitrophenol isomers in Table 1.

**TABLE 1:** Comparison between the determined pKa values for guaiacols with the literature values of phenols; phenol (PhOH), *m*-nitrophenol (*m*-NP), *p*-nitrophenol (*p*-NP), and 2,4-dinitrophenol (DNP).

Compound	pKa (experimental)	pKa (literature)
4NG	6.4	
5NG	8.5	
46DNG	3.3	
GUA	10.0	9.98^a^
PhOH		10.0^b^
*m*-NP		8.4^b^
*p*-NP		7.2^b^
DNP		4.1^b^

^a^ Varekova et al. (2011).

^b^ Dewick (2013).

The determined pKa of GUA corresponds very well to the estimated one by use of quantum chemical calculations (Varekova et al., 2011). Moreover, the order of pKa values for phenol and its nitro derivatives is similar to the one we obtained for GUA and NGs. While the pKa values for phenol and GUA as well as for *m*-NP and 5NG pairs are practically the same, somehow lower pKa were determined for 4NG and 46DNG compared to *p*-NP and 2,4-DNP, respectively. These discrepancies can be attributed to the electron-donating methoxy substituent in the case of NGs.

We can conclude from these data that >90% of 4NG and 5NG were in their protonated forms during the photolysis experiments, which were performed at pH 5 as a proxy of atmospheric aqueous phase. On the other hand, the formed 46DNG was mostly ionized during the experiments and could serve as a source of additional hydronium ions in the photolyzed 4NG solutions (see the text below).

### 3.2 Photolysis experiments

#### 3.2.1 Spectrophotometric analysis

**FIGURE 2:**
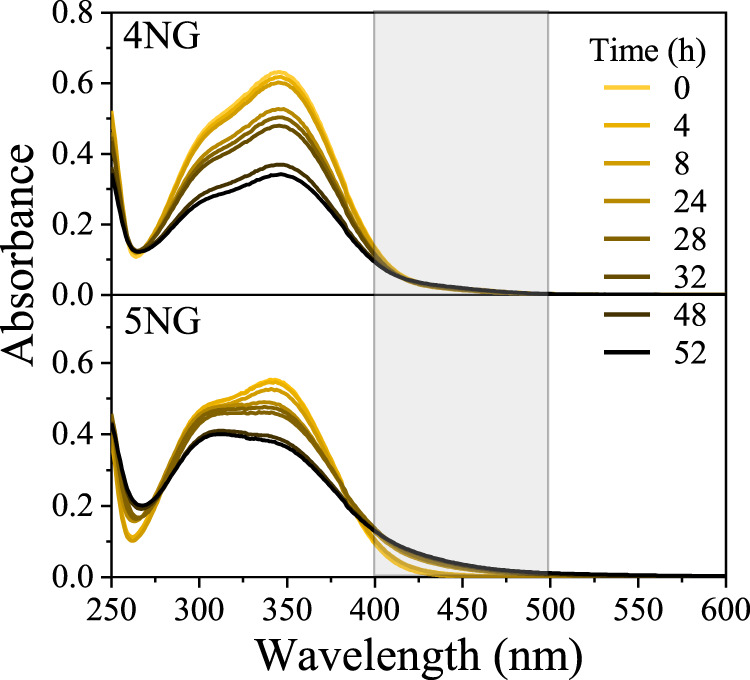
Absorption spectra of 4NG and 5NG photolysis solutions at different exposure times.

By measuring absorption spectra of the reaction mixtures in a timeframe of 52 h, we observed a decrease in absorbance of the characteristic bands for both 5NG and 4NG (Figure 2), implying original molecule degradation.

From these data it seems that the photolysis of 5NG progressed slower than that of 4NG, which is indicated by a lesser decrease in the characteristic absorbance band at 350 nm (Figure 2). Moreover, the measured spectra also imply that artificial sunlight significantly altered chemical composition of the reaction mixtures, which is evident from the gradual increase of absorbance at longer wavelengths for both investigated compounds that can be attributed to the formation of vis-absorbing products (>400 nm; marked with a gray box in Figure 2). Oppositely to the slower disappearance of the original molecular band, however, these products prevailed in the case of 5NG (there were either chromophores formed in larger amounts or those formed were more intensively colored), which was also confirmed by observing a more intense yellowish-brown color of the reaction mixture at the end of the experiment. Similar absorption spectra, including in the visible range, were characteristic of the simulated atmospheric aerosol from GUA in the high NOx environment, particularly when formed at high relative humidity (Kroflič et al., 2021). At the same time, the UVC region of the 5NG-illuminated spectra also changed significantly (note the isosbestic point at 278 nm), whereas the spectra of 4NG practically retained their original shape in this wavelength range. This part of the spectra, however, is not relevant for the atmosphere because the majority of light below 300 nm is absorbed by the stratospheric ozone anyways.

#### 3.2.2 Product studies

For the product analyses we chose the samples at the end of each experiment, which eventually contained the highest quantity of the formed products with retained aromaticity. Based on the comparison of recorded HPLC-DAD spectra with standards, we found that GUA was absent in all reaction mixtures (or was below the detection limit), although it was expected to be formed in analogy with the *p*-NP photolysis (Chen et al., 2005). The obtained chromatographic peaks were further investigated by LC-MS/MS. Detailed product identification was performed by studying MS/MS fragmentation patterns of the identified deprotonated molecular ions [ M−H ]^−^, which were compared with standards whenever possible. Fragmentation patterns of unknown compounds showed characteristic neutral losses of NO (−30), OH (−17), and sometimes CH_3_ (−15) and CO (−28), pointing to the presence of aromatic nitro, methoxy, and hydroxy groups (Frka et al., 2016). Characteristic SRM transitions are collected in Supplementary Table S4.

**FIGURE 3:**
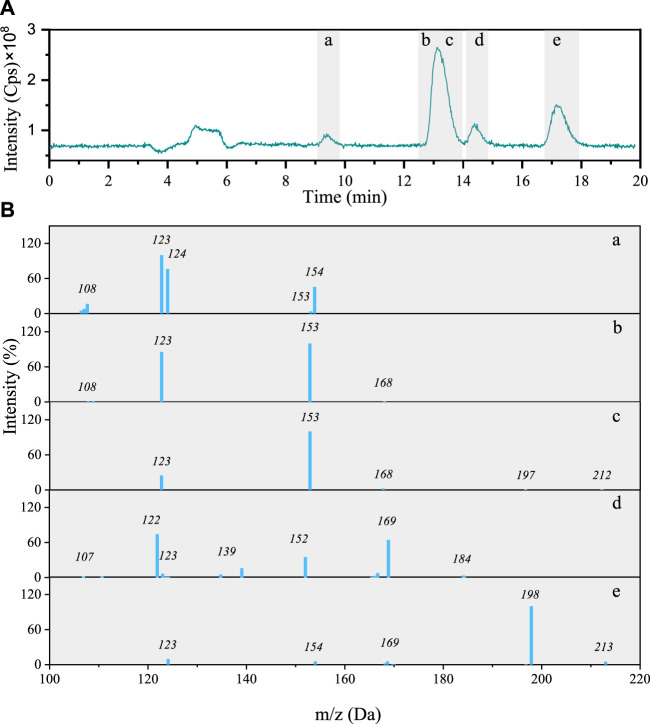
**(A)** Total ion chromatogram after 48 h photolysis of 4NG along with **(B)** MS/MS spectra of the separated compounds (a–e).

Total ion chromatogram (TIC) of the 4NG photolyzed solution contains four peaks including five different components with characteristic molecular ions: *m/z* 213, 212, 184, 154, and 168 (Figure 3A). The identified molecular ions corresponding to peaks A–E were subjected to further fragmentation and the results are shown in Figure 3B.

The fragmentation pattern of *m/z* 154 eluted at 9 min (Figures 3B-b) shows characteristic neutral losses of NO (*m*/*z* 124) and OH (*m/z* 107), which fit with 4-nitrocatehol (4NC). Peak B found at the retention time (RT) of 14 min (molecular ion *m/z* 168) agrees with 4NG with the molar mass of 169 Da (Figures 3B-b). The identity of both compounds was confirmed by comparison with the corresponding standard compounds.

Although the compounds with *m/z* 212 (Figures 3B-c) and 184 (Figures 3B-d) could not be unequivocally confirmed, their structures are tentatively proposed in Supplementary Table S1 (refer here to compounds number 7 and 6, respectively). The fragmentation pattern of *m/z* 212 possibly points to the presence of the carboxylic group due to the neutral loss characteristic of CO_2_ (−44) giving *m/z* 168 fragment.

The *m/z* 213 compound elutes at 17 min and from its fragmentation pattern (Figures 3B-e) neutral losses of CH_3_ (*m/z* 154) and NO (*m/z* 124) could be identified, which agree with 46DNG. The identity of this compound was again additionally confirmed by comparison of its RT and MS/MS spectrum with the standard compound. As the acidity of 46DNG is stronger than that of the precursor molecules, this reaction pathway resulted in the concomitant release of H_3_O^+^ ions, which was confirmed by a drop in solution pH by the end of experiment (refer here to Supplementary Table S2). Furthermore, the drop in solution pH could also result from the nitrous acid release (Chen et al., 2005), which cannot be confirmed for our experimental system as it was not specifically targeted in this study. Moreover, there seem to be even more products eluted from the column soon after the solvent front (around RT = 5 min, Figure 3A), which we were unable to separate and identify with the applied methods.

**FIGURE 4:**
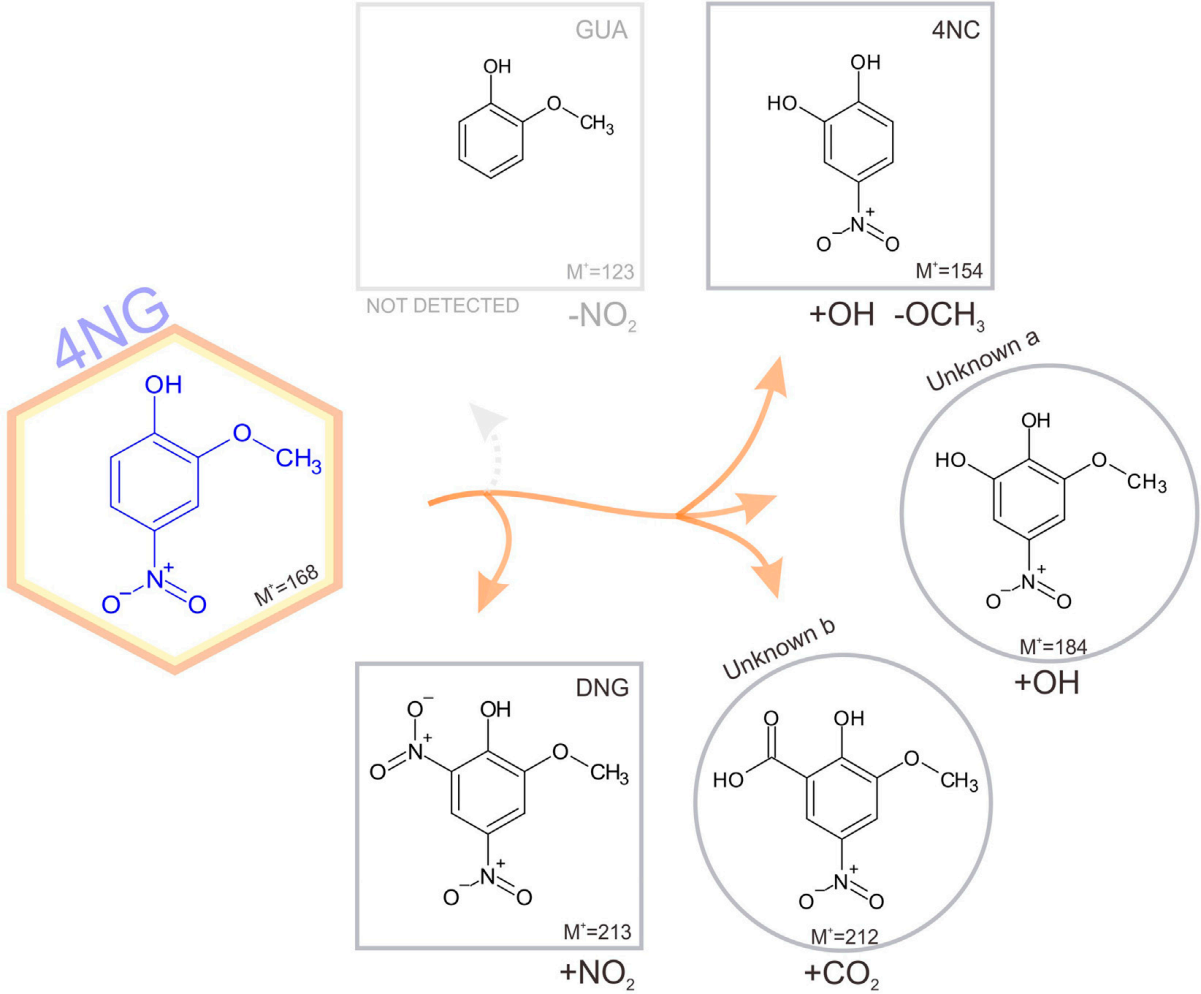
Schematic representation of identified pathways of 4NG photo-catalyzed transformations in an illuminated aqueous solution. Products shown in square boxes were confirmed by comparison with commercial standards; in round boxes, tentative structures are proposed based on the measured fragmentation patterns. Note, GUA (or its non-nitrated derivatives) were not detected, but must have been formed. The corresponding mechanisms are proposed in the text.

The identified photodegradation pathways of water-dissolved 4NG under artificial sunlight are schematically represented in Figure 4.

Poor sensitivity of the HPLC analysis and the lack of ionization of non-nitrated phenols in the ESI source (e.g., GUA and other hydroxylated benzenes) prevented us from unequivocally confirming the photo-induced loss of the nitro group from the aromatic ring (note the dotted arrow for the pathway to GUA formation), which was previously observed by Chen and co-workers (2005) in the case of *p*-NP yielding phenol upon photolysis. On the other hand, intermediate denitration products, such as phenoxy radicals as proposed by other authors might have been formed as well (Barsotti et al., 2017), allowing for a plethora of possible final products, including oligomer formation, which were again out of reach of the implemented methods. Nevertheless, reactive nitrogen species (NO^•^, NO_2_^•^ and/or HONO) must have been formed in the solution yielding the detected 46DNG product in the subsequent reaction steps, which could only originate from 4NG as this was the only source of nitrogen added to the solutions. The most likely mechanism leading to the observed dinitrated product is through the formation of an [aromatic-OH]^•^ adduct and consequent water elimination to the corresponding phenoxy radical, followed by NO_2_^•^ addition to 46DNG (Kroflič et al., 2018). This, however, further implies that hydroxyl radicals were also present in the solutions and were either a direct product of 4NG photolysis (Guo and Li, 2022) or were secondarily formed in the solution by the photolysis of released reactive nitrogen species, such as NO_2_^•^ and HONO.

Products containing more than one hydroxyl group were also detected, similar to study of Chen and co-workers (Chen et al., 2005), and support the involvement of secondary ^•^OH chemistry in the investigated systems (refer here to the Unknown a in Figure 4). Phenolic hydroxylation in aqueous solutions is mostly described by ^•^OH addition to the aromatic ring and consecutive reaction with O_2_, followed by HO_2_^•^ elimination to the corresponding hydroxyphenol (Barzaghi and Herrmann, 2002). Moreover, *ipso*-attack of ^•^OH to the methoxy group-bearing C-atom followed by methanol elimination is proposed as the main mechanism of catechol formation from guaiacol (Aihara et al., 1993), which has also been observed in a multi-phase system as a proxy for atmospheric aerosols at high relative humidity (Kroflič et al., 2021). On the other hand, the minor demethoxylation route starting with ^•^OH-assisted hydrogen abstraction and resulting in formaldehyde abstraction (Aihara et al., 1993) could lead to the Unknown b formation through the acid-catalyzed 4NG and formaldehyde condensation followed by the oxidation in an oxygen atmosphere or by HONO. The reaction between formaldehyde and phenol, however, has never been confirmed under mild atmospheric conditions.

**FIGURE 5:**
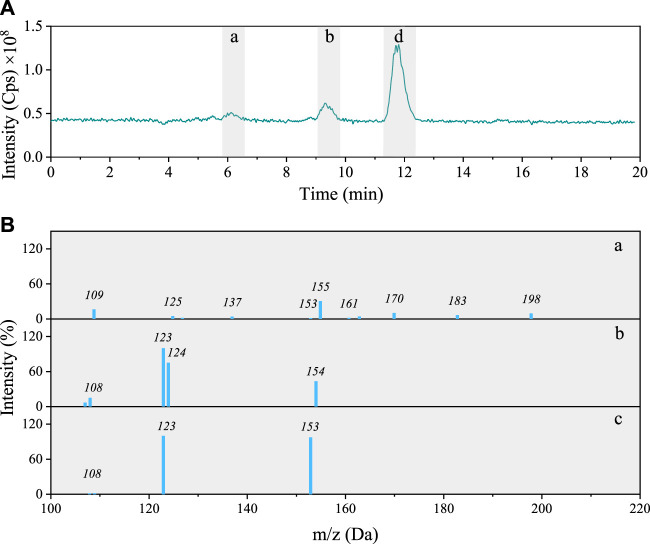
**(A)** Total ion chromatogram after 48 h photolysis of 5NG along with **(B)** MS/MS spectra of the separated compounds (a–c).

Total ion chromatogram of the 5NG photolyzed solution shows only three distinct peaks (Figure 5A) with [ M−H ]^−^ molecular ions of m/z 198, 154, and 168, which were again subjected to further fragmentation as presented in Figure 5B.

The characteristic of the first chromatographic peak in Figure 5B is the molecular ion *m/z* 198, its fragmentation yielding fragments at *m/z* 183 (−15; neutral loss of CH_3_), *m/z* 155 (−28; loss of CO) and *m/z* 125 (−30; consecutive neutral loss of NO). Its signal, however, is too small to allow for the identification with certainty. A possible molecular structure of the corresponding compound based on the MS/MS data alone is shown in Supplementary Table S1 (compound 8).

The peak at 9 min on the other hand corresponds to the same species also found in the 4NG sample, i.e., 4NC (compare fragmentation patterns in Figures 3A-a; Figures 5B-b). The peak at RT 11.7 min agrees with 5NG (Figures 5B-C), its fragmentation showing neutral losses of CH_3_ (*m/z* 153) and NO (*m/z* 123), which was also confirmed by comparison with the standard compound.

**FIGURE 6:**
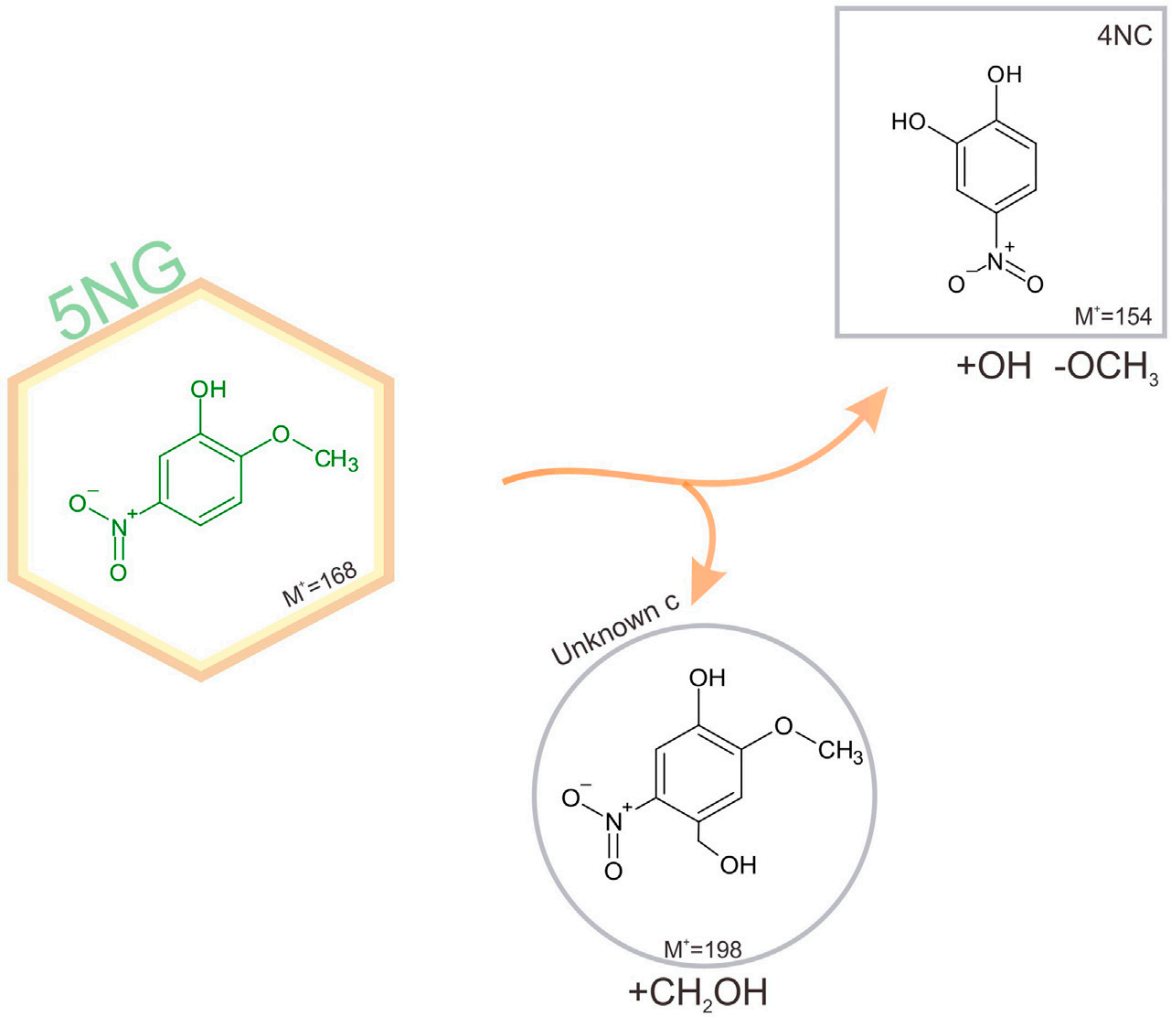
Schematic representation of identified pathways of 5NG photo-catalyzed transformations in an illuminated aqueous solution. Products shown in square boxes were confirmed by comparison with commercial standards; in round boxes, tentative structures are proposed based on the measured fragmentation patterns. The corresponding mechanisms are proposed in the text.

Schematic representation of 5NG photodegradation pathways is shown in Figure 6.

Lack of evidence for the possible 5NG denitration is in line with the previous study on *m*-NP photolysis (Chen et al., 2005). Again, the demetoxylated product (4NC) was observed, which must have involved the ^•^OH chemistry as explained above. Moreover, similar to the industrial acid-catalyzed synthesis of phenol-formaldehyde resins (Lu et al., 2004), the Unknown c could be formed as a precursor to the carboxylated analogue observed in the 4NG solutions, stopping at this stage due to lower concentrations of oxidant species in the solution (e.g., HONO) as a result of slower photolysis rate. As already mentioned, this is only speculation and would need to be confirmed under atmospheric conditions.

As we previosly showed that the absorption spectra of 5NG photolysed solutions changed more significantly than those of 4NG photolysed solutions, we can now attribute those changes to a small number of photoproducts with retained aromaticity and nitro substitution, or oligomer formation. On the other hand, in the case of 4NG photolysis, the more numerous products obviously possess comparable spectroscopic properties to the parent compound (or do not absorb in the investigated range at all), resulting in very limited changes in the absorption spectrum after the photolysis, except for the altered peak intensity. This agrees with the proposed formation of non-nitrated phenols, which could not be unequivocally confirmed in our study, but must have been formed based on the evidence given above.

### 3.3 Kinetics

**FIGURE 7:**
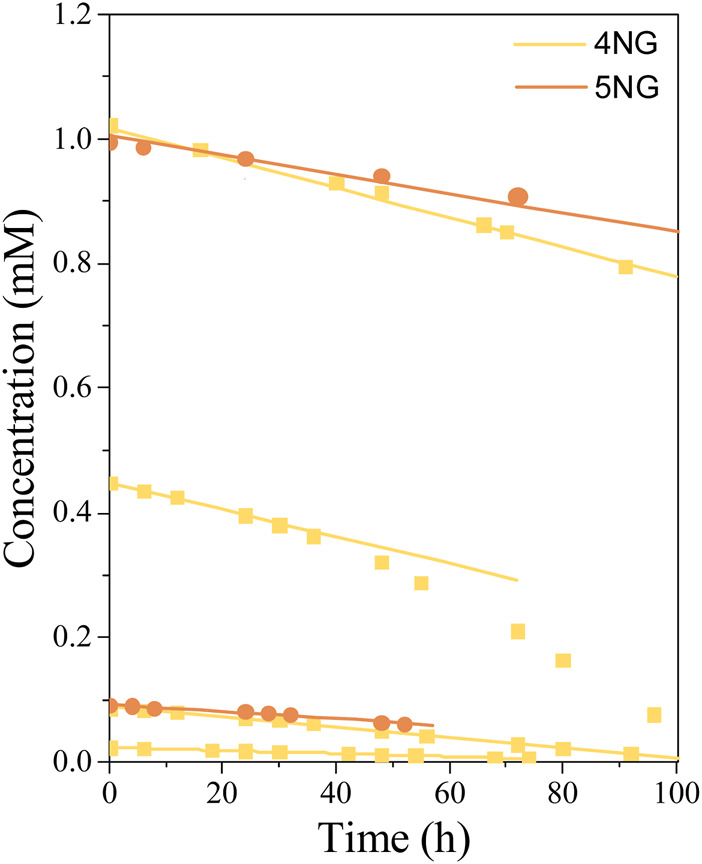
Concentration profiles of 4NG and 5NG photolysis at different initial concentrations during illumination with the artificial sunlight; symbols represent experimental data, while lines are linear fits through the (initial) experimental points.

Figure 7 shows concentration changes during the photolysis of 4NG and 5NG containing aqueous solutions with time. At the same initial concentration, 5NG concentration decreased slower than the concentration of 4NG, which agrees with the spectroscopic data presented above and also with the studies performed on the nitrophenol isomers (Chen et al., 2005).

Most experimental data could be well described by linear functions (*r* ^2^ ≥ 0.97), which means that during the photolysis experiments, changing reactant concentration had no effect on the reaction rate itself. Similarly, zero-order photolysis kinetics was also observed in the case of nitrophenol photolysis (Chen et al., 2005). In one specific case, however, the reaction rate increased after some time (0.45 mM 4NG solution; Figure 7), probably due to the formation of photosensitizer products (Felber et al., 2021), which are out of scope of this study.

The photolysis kinetics was evaluated by the initial rate method. Linear regression was used to estimate the reaction rates at the beginning of each experiment, while according to the zero-order kinetics, the slope of the line (i.e., the reaction rate) equals the kinetic rate constant. For most experimental conditions (0.025, 0.1, and 1.0 mM initial concentrations), these were representative reaction rate constants throughout the experiments, whereas the experiment at 0.45 mM 4NG showed some deviation from linearity, which was already mentioned before. The resulting zero-order kinetic parameters (Eq. 5) are gathered in Table 2. Our photolysis rates under the artificial sunlight are for two orders of magnitude lower than those determined for 0.1 mM solutions of *p*-NP and *m*-NP under the Mercury lamp, i.e. 0.0486 mM/h and 0.0151 mM/h, respectively (Chen et al., 2005).

**TABLE 2:** Kinetic parameters for the photolysis of 4NG (1–4) and 5NG (5–6) in aqueous solutions irradiated with the artificial sunlight.

	Init. conc. (mM)	Reaction rate (mM/h)	*r* ^2^	Rate constant	Reaction order	Lifetime (h)
1	1.0	−0.0024	0.97	−0.002 mM/h	zero	—
2	0.45	−0.0022^a^	0.99	−0.005 h^−1^ ^a^	cannot be determined	200^a^
3	0.1	−0.0008	0.99	−0.008 h^−1^	pseudo-zero	125
4	0.025	−0.0002	0.99	−0.008 h^−1^	pseudo-zero	125
5	1.0	−0.0015	0.98	−0.0015 mM/h	zero	—
6	0.1	−0.0006	0.99	−0.006 h^−1^	pseudo-zero	167

^a^ Overestimated, see discussion.

**FIGURE 8:**
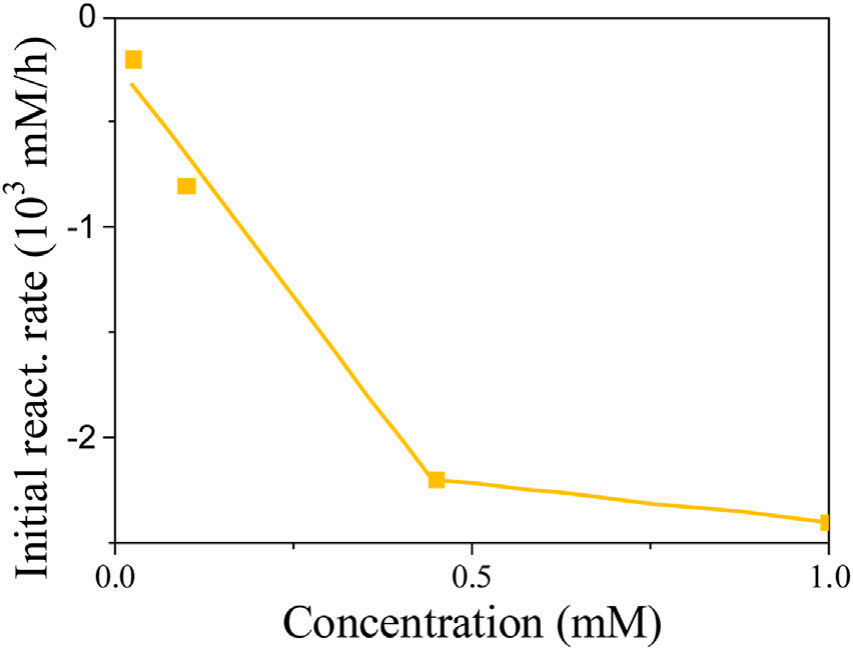
Initial reaction rates for different starting 4NG concentrations.

Based on the linearity of response, one would easily conclude that 4NG photolysis is of zero-order kinetics and only depends on the photon flux to the solution. However, different slopes for different experiments in Figure 7 suggest that the kinetics is of pseudo-zero order and that real reaction rate also depends on initial reactant concentration, which is demonstrated in Figure 8.

We found out at low reactant concentrations that the determined reaction rates are directly proportional to the corresponding reactant concentration at the beginning of each experiment, which agrees with the first-order kinetics. However, the slopes at 0.45 and 1.00 mM 4NG became equal, implying that the initial reactant concentration indeed ceases to have an effect on the reaction rate at high reactant concentrations, following real zero-order kinetics at those conditions.

Nevertheless, considering the effect of initial reactant concentration on the observed reaction rates, 4NG photolysis is of pseudo-zero order at low concentrations (<0.45 mM), the slope of the line indicating an apparent rate constant of the zero order ( $k_{app}={k_{0}=k}_{1}∙c_{0}$ ). Therefore, we were able to calculate the corresponding first-order rate constants for the three experimental systems (Table 2) and to further predict photolysis lifetimes in the atmospheric waters according to the Eq. 7. For the reaction regime of the lowest two concentrations, the lifetime was estimated at 125 h. However, the actual lifetime for 0.45 mM 4NG estimated at 200 h should be much shorter due to photosensitation reactions taking place in the solution. For concentrations higher than this, it is impossible to calculate a unique lifetime as it depends on the reactant concentration itself as explained above.

Based on the 4NG photolysis results, photodegradation of 5NG is also expected to be of the pseudo-zero order at low reactant concentrations and of real zero order at high initial concentrations, although the borderline concentration for the change of the regime is not evident from our experimental data. We thus only estimated the lifetime for the lowest 0.1 mM concentration, which is with 167 h longer than that of 4NG, in line with our previous discussion.

Both atmospheric lifetimes are much longer than those observed for the gas-phase photolysis of NPs, which were determined in the order of minutes (Peng et al., 2023).

### 3.4 Atmospheric applications

The determined pKa values of the investigated compounds and their major photodegradation products were in the following order: 46DNG < 4NG < 5NG < GUA. Although we found light absorption by all investigated compounds is pH-dependent, 4NG and 5NG are expected to mostly contribute to the atmospheric light absorption by BrC under basic conditions, which is rarely relevant for the atmospheric aqueous phase. In contrast, 46DNG significantly absorbs visible light in a wide range of pH values (especially above pH 3.3) and is expected to respond to the changing secondary aerosol pH with changing atmospheric conditions. Acidic aqueous phase (pH < 5) is typical for liquid aerosols and cloud water in rural areas and polluted urban environments, whereas higher pH values (pH > 4) are more characteristic of atmospheric waters in very clean environments, expecting to enhance the atmosphere-heating effect of long-range transported NG aerosols in these sensitive regions.

We further found the photolysis of aqueous-phase 4NG under the artificial sunlight faster than that of 5NG; however, this was not resembled in the visible part of the recorded absorption spectra. More visible absorption was observed in the case of 5NG photolysis products at the end of experiments, implying its higher potential to contribute to atmospheric light absorption by secondary BrC aerosols.

The kinetics of 4NG and 5NG photolysis was of the pseudo-first order at low precursor concentrations and changed to the real zero-order above some marginal initial concentration (around 0.45 mM), which is expected to change upon addition of other light-absorbing species competing for the incoming photons with the target compounds. This, however, needs to be confirmed in future studies. Moreover, the effect of photosensitation was also observed that was out of scope of this study. At pH 5, atmospheric lifetimes of aqueous-phase 4NG and 5NG were determined at 125 and 167 h, respectively, which implies their potential damaging influence on the distant regions downwind from the emission sources. Such long atmospheric lifetimes also suggest 4NG and 5NG can be used as biomass-burning marker compounds.

The photolysis of both precursor molecules (4NG and 5NG) yielded photoproducts with preserved aromatic ring, –OCH_3_, –OH, and–NO_2_ groups. Based on the confirmed presence of 46DNG, which formed during the 4NG photolysis, the nitro group must have been detached from the 4NG aromatic ring (in a form of NO_2_^•^ and/or HONO) and reacted further with another 4NG molecule. Chemical transformations involving secondary ^•^OH are further proposed, additionally pointing to the influence of both, direct and indirect photodegradation pathways to the observed precursor decays, involving also reactive species that formed in the solutions during the experiment. In short, denitration, carbon loss, hydroxylation, nitration, and carbon gain were characteristic of 4NG phototransformation, while carbon loss, hydroxylation, and carbon gain were observed in the case of 5NG. Although mechanisms underlying the observed reaction pathways are discussed based on the existing literature, these still need to be confirmed in future laboratory and field studies.

## Data Availability

The original contributions presented in the study are included in the article/Supplementary Material, further inquiries can be directed to the corresponding authors.
