# Supplementary material for: Kinetics and product identification of water-dissolved nitroguaiacol photolysis under artificial sunlight
Source: Front Chem. 2023 Jul 14;11:1211061. doi: 10.3389/fchem.2023.1211061 (PMC10375238; doi:10.3389/fchem.2023.1211061)
Supplement: Supplementary file 1 [file DataSheet1.docx]

# Supporting Information for

# Kinetics and product identification of water-dissolved nitroguaiacol photolysis under artificial sunlight

Ajda Delić,^1,2^ Urša Skube,^2^ Martin Šala,^2,^* and Ana Kroflič^1,2,^*

^1^Department of Catalysis and Chemical Reaction Engineering, National Institute of Chemistry, Ljubljana, Slovenia
^2^Department of Analytical Chemistry, National Institute of Chemistry, Ljubljana, Slovenia

Corresponding Authors:
Ana Kroflič* ana.kroflic@ki.si
Martin Šala* martin.sala@ki.si

Table S1: Investigated compounds and their chemical properties; 1–5 are the identified photolysis products by comparison with standards, 6–8 are detected photolysis products with tentative structures proposed.

|  | Compound | Molecular formula | Structural formula | M (g/mol) | [M-H]^-^ (Da) |
| --- | --- | --- | --- | --- | --- |
| 1 | GUA  guaiacol  2-methoxyphenol  CAS: 90-05-1 | C_7_H_8_O_2_ |  | 124.14 | 123 |
| 2 | 4NG  4-nitroguaiacol  2-methoxy-4-nitrophenol  CAS: 3251-56-7 | C_7_H_7_NO_4_ |  | 169.13 | 168 |
| 3 | 5NG  5-nitroguaiacol  2-methoxy-5-nitrophenol  CAS: 636-93-1 | C_7_H_7_NO_4_ |  | 169.13 | 168 |
| 4 | 46DNG  4,6-dinitroguaiacol  2-methoxy-4,6-dinitrophenol  CAS: 4097-63-6 | C_7_H_6_N_2_O_6_ |  | 214.13 | 213 |
| 5 | 4NC  4-nitrocatechol  4-nitrobenzene-1,2-diol  CAS: 3316-09-4 | C_6_H_5_NO_4_ |  | 155.11 | 154 |
| 6 | Unknown a | C_7_H_7_NO_5_ |  | 185.13 | 184 |
| 7 | Unknown b | C_8_H_7_NO_6_ |  | 213.14 | 212 |
| 8 | Unknown c | C_8_H_9_NO_5_ |  | 199.16 | 198 |

Table S2: Solution pH values as measured at the beginning and at the end of each experiment.

| Concentration [mM] | Start pH | End pH |
| --- | --- | --- |
| 4NG | | |
| 1 | 4.5 | 3.6 |
| 0.45 | 4.9 | 3.4 |
| 0.1 | 5.0 | 4.0 |
| 0.025 | 5.0 | 4.5 |
| 5NG | | |
| 1 | 5.0 | 3.7 |
| 0.1 | 5.0 | 4.4 |

Table S3: Conditions used for the determination of the pKa values of guaiacol and nitroguaiacols.

| Compound | Concentration  [mM] | Characteristic wavelength [nm] | Investigated pH range |
| --- | --- | --- | --- |
| 4NG | 0.08 | 430 | 4–8 |
| 5NG | 0.08 | 430 | 5–10 |
| 46DNG | 0.045 | 380 | 2–7 |
| GUA | 0.16 | 295 | 5–11 |

Table S4: SRM parameters (transitions) used in HPLC-(−)ESI-MS/MS analysis; DP stands for declustering potential and CE is collision energy.

| Compound | Q1 *m/z* | Q3 *m/z* | Acquisition time (ms) | DP (V) | CE (V) |
| --- | --- | --- | --- | --- | --- |
| 4NG, 5NG | 168 | 153 | 100 | −44 | −20 |
| 46DNG | 213 | 198 | 100 | −50 | −30 |
| 4NC | 154 | 124 | 100 | −80 | −20 |
| Unknown a | 184 | 169 | 100 | −50 | −22 |
| Unknown b | 212 | 153 | 100 | −45 | −22 |
| Unknown c | 198 | 154 | 100 | −50 | −30 |

Figure S1: Emission spectra of the Xenon lamp measured at the specified distance from the light source above and under the glass of the reactor flask together with the absorption spectra of 4N and 5N.

Figure S2: Chromatograms of blank experiments: A) 0.1 mM 4NG and B) 0.1 mM 5NG ageing in the dark for 48 h.

Figure S3: Absorbance dependence at the selected wavelengths on the solution pH for GUA (orange, 295 nm), 5NG (pink, 430 nm), 4NG (gray, 430 nm), and 46DNG (dark purple, 380 nm). Measured values are represented with dots and modeled with solid lines.
